# National health policies and strategies for addressing chronic kidney disease: Data from the International Society of Nephrology Global Kidney Health Atlas

**DOI:** 10.1371/journal.pgph.0001467

**Published:** 2023-02-01

**Authors:** Brendon L. Neuen, Aminu K. Bello, Adeera Levin, Meaghan Lunney, Mohamed A. Osman, Feng Ye, Gloria E. Ashuntantang, Ezequiel Bellorin-Font, Mohammed Benghanem Gharbi, Sara Davison, Mohammad Ghnaimat, Paul Harden, Vivekanand Jha, Kamyar Kalantar-Zadeh, Peter G. Kerr, Scott Klarenbach, Csaba P. Kovesdy, Valerie Luyckx, Shahrzad Ossareh, Jeffrey Perl, Harun Ur Rashid, Eric Rondeau, Emily J. See, Syed Saad, Laura Sola, Irma Tchokhonelidze, Vladimir Tesar, Kriang Tungsanga, Rumeyza Turan Kazancioglu, Angela Yee-Moon Wang, Chih-Wei Yang, Alexander Zemchenkov, Ming-hui Zhao, Kitty J. Jager, Fergus J. Caskey, Vlado Perkovic, Kailash K. Jindal, Ikechi G. Okpechi, Marcello Tonelli, John Feehally, David C. Harris, David W. Johnson

**Affiliations:** 1 The George Institute for Global Health, University of New South Wales, Sydney, Australia; 2 Department of Renal Medicine, Royal North Shore Hospital, Sydney, Australia; 3 Division of Nephrology and Immunology, Department of Medicine, University of Alberta, Edmonton, Canada; 4 Division of Nephrology, Department of Medicine, University of British Columbia, Vancouver, Canada; 5 Department of Community Health Sciences, University of Calgary, Calgary, Canada; 6 Faculty of Medicine and Biomedical Sciences, Yaounde General Hospital, University of Yaounde, Yaounde, Cameroon; 7 Division of Nephology and Hypertension, Department of Medicine, Saint Louis University, Saint Louis, Missouri, United States of America; 8 Urinary Tract Diseases Department, Faculty of Medicine and Pharmacy of Casablanca, University Hassan II of Casablanca, Casablanca, Morocco; 9 Nephrology Division, Department of Internal Medicine, The Specialty Hospital, Amman, Jordan; 10 Oxford Kidney Unit, Oxford University Hospitals NHS Foundation Trust, Oxford, United Kingdom; 11 George Institute for Global Health India, New Delhi, India; 12 School of Public Health, Imperial College, London, United Kingdom; 13 Manipal Academy of Higher Education, Manipal, India; 14 Division of Nephrology and Hypertension, University of California Irvine Medical Center, Orange, California, United States of America; 15 Department of Medicine, Monash University, Melbourne, Australia; 16 University of Tennessee Health Science Center, Memphis, Tennessee, United States of America; 17 Department of Paediatrics and Child Health, University of Cape Town, Cape Town, South Africa; 18 Renal Division, Brigham and Women’s Hospital, Harvard Medical School, Boston, Massachusetts, United States of America; 19 Division of Nephrology, Department of Medicine, Hasheminejad Kidney Center, Iran University of Medical Sciences, Tehran, Iran; 20 Division of Nephrology, St. Michael’s Hospital and the Keenan Research Centre in the Li Ka Shing Knowledge Institute, St. Michael’s Hospital, Toronto, Canada; 21 Division of Nephrology, Department of Medicine, University of Toronto, Toronto, Canada; 22 Department of Nephrology, Kidney Foundation Hospital and Research Institute, Dhaka, Bangladesh; 23 Intensive Care Nephrology and Transplantation Department, Hopital Tenon, Assistance Publique-Hopitaux de Paris, Paris, France; 24 Sorbonne Université, Paris, France; 25 Department of Intensive Care, Austin Health, Melbourne, Australia; 26 School of Medicine, University of Melbourne, Melbourne, Australia; 27 Dialysis Unit, CASMU-IAMPP, Montevideo, Uruguay; 28 Nephrology Development Clinical Center, Tbilisi State Medical University, Tbilisi, Georgia; 29 Department of Nephrology, General University Hospital, Charles University, Prague, Czech Republic; 30 Department of Medicine, Faculty of Medicine, King Chulalongkorn Memorial Hospital, Chulalongkorn University, Bangkok, Thailand; 31 Division of Nephrology, Bezmialem Vakif University, Istanbul, Turkey; 32 Department of Medicine, Queen Mary Hospital, The University of Hong Kong, Pok Fu Lam, Hong Kong; 33 Kidney Research Center, Department of Nephrology, Chang Gung Memorial Hospital, Chang Gung University College of Medicine, Taoyuan, Taiwan; 34 Department of Internal Disease and Nephrology, North-Western State Medical University named after I.I. Mechnikov, Saint Petersburg, Russia; 35 Department of Nephrology and Dialysis, Pavlov First Saint Petersburg State Medical University, Saint Petersburg, Russia; 36 Renal Division, Department of Medicine, Peking University First Hospital, Beijing, China; 37 Key Lab of Renal Disease, Ministry of Health of China, Beijing, China; 38 Key Lab of Chronic Kidney Disease Prevention and Treatment, Ministry of Education of China; Beijing, China; 39 Peking-Tsinghua Center for Life Sciences, Beijing, China; 40 ERA Registry, Department of Medical Informatics, Amsterdam Public Health Research Institute, Amsterdam UMC, University of Amsterdam, Amsterdam, The Netherlands; 41 Population Health Sciences, University of Bristol, Bristol, United Kingdom; 42 The Richard Bright Renal Unit, Southmead Hospital, North Bristol NHS Trust, Bristol, United Kingdom; 43 Division of Nephrology and Hypertension, University of Cape Town, Cape Town, South Africa; 44 Kidney and Hypertension Research Unit, University of Cape Town, Cape Town, South Africa; 45 Department of Medicine, University of Calgary, Calgary, Canada; 46 Pan-American Health Organization/World Health Organization’s Collaborating Centre in Prevention and Control of Chronic Kidney Disease, University of Calgary, Calgary, Canada; 47 University of Leicester, Leicester, United Kingdom; 48 Centre for Transplantation and Renal Research, Westmead Institute for Medical Research, University of Sydney, Sydney, Australia; 49 Centre for Kidney Disease Research, University of Queensland, Brisbane, Australia; 50 Translational Research Institute, Brisbane, Australia; 51 Metro South and Ipswich Nephrology and Transplant Services (MINTS), Princess Alexandra Hospital, Brisbane, Australia; Sree Chitra Tirunal Institute for Medical Sciences and Technology, INDIA

## Abstract

National strategies for addressing chronic kidney disease (CKD) are crucial to improving kidney health. We sought to describe country-level variations in non-communicable disease (NCD) strategies and CKD-specific policies across different regions and income levels worldwide. The International Society of Nephrology Global Kidney Health Atlas (GKHA) was a multinational cross-sectional survey conducted between July and October 2018. Responses from key opinion leaders in each country regarding national NCD strategies, the presence and scope of CKD-specific policies, and government recognition of CKD as a health priority were described overall and according to region and income level. 160 countries participated in the GKHA survey, comprising 97.8% of the world’s population. Seventy-four (47%) countries had an established national NCD strategy, and 53 (34%) countries reported the existence of CKD-specific policies, with substantial variation across regions and income levels. Where CKD-specific policies existed, non-dialysis CKD care was variably addressed. 79 (51%) countries identified government recognition of CKD as a health priority. Low- and low-middle income countries were less likely to have strategies and policies for addressing CKD and have governments which recognise it as a health priority. The existence of CKD-specific policies, and a national NCD strategy more broadly, varied substantially across different regions around the world but was overall suboptimal, with major discrepancies between the burden of CKD in many countries and governmental recognition of CKD as a health priority. Greater recognition of CKD within national health policy is critical to improving kidney healthcare globally.

## Introduction

Chronic kidney disease (CKD) is a major global health issue. Estimates indicate that almost 10% of the world’s population are affected by some form of CKD accounting for 1.2 million deaths annually [1]. It is projected that by 2040, CKD will be the 5^th^ leading cause of years of life lost globally–one of the largest projected increases of any major noncommunicable disease (NCD) [2]. In many regions around the world, including Central Latin America, Sub-Saharan Africa and Central Asia, growth in the burden of CKD due to diabetes and hypertension is outpacing that of population growth and aging, with low- and middle-income countries disproportionately affected [3].

The large and growing global burden of CKD has far reaching implications for individuals and health systems. Most people with CKD die prematurely due to cardiovascular disease before reaching kidney failure, with poorer outcomes observed especially for those with diabetes [4]. CKD is therefore a ‘risk multiplier’ for other priority NCDs, particularly cardiovascular disease. Because of the increasing global prevalence of CKD, the number of people projected to require kidney replacement therapy in the form of dialysis or kidney transplantation is also projected to increase. About 2.6 million people were estimated to have received dialysis or undergone kidney transplantation for kidney failure in 2010, and this number is projected to more than double by 2030 [5]. Notwithstanding impacts on patients, families and their caregivers, the growing burden of CKD has major economic implications, given the high cost of providing kidney care, especially dialysis and transplant services [6].

These realities highlight the importance of recognizing and prioritizing CKD care through a broader NCD strategy and through CKD-specific policies. Previously however, CKD has not been given the priority it deserves as a cause, consequence and risk multiplier of many other priority NCDs. The United Nations Sustainable Development Goals (SDGs) have set a target for reducing premature mortality from priority NCDs by a third by 2030 [7]. Whilst CKD is not formally addressed, there is increasing recognition that targeting shared risk factors of priority NCDs such as diabetes and cardiovascular disease, as well as addressing other SDGs relevant to health have the potential to accelerate progress towards reducing the global burden of CKD [8].

The International Society of Nephrology (ISN) Global Kidney Health Atlas (GKHA), now in its second iteration, sought to provide important information on CKD risk factors, the burden and consequences of CKD, and gaps in specific kidney care areas in different countries around the world [9]. The ISN GKHA also collected granular data on national strategies and policies for CKD care, with a view to benchmarking capacity to deliver kidney healthcare and serving as an advocacy tool for health system improvement.

We herein report on national NCD and CKD-specific strategies and policies for addressing CKD around the world to better understand variations across regions and country income levels.

## Methods

The second iteration of the GKHA was a cross-sectional survey conducted by the International Society of Nephrology (ISN). Detailed descriptions of the sampling approach, survey development, data handling, statistical analysis, and main results have been previously published [10, 11]. Briefly, two approaches were used to gather the data for the study: desk research and an online survey of key stakeholders from each participating country.

### Desk research

We performed a comprehensive review of the literature with an information specialist to synthesize national health policies and strategies for addressing CKD. Data were collected from government reports, national registries, and published as well as grey literature. This included data from the World Health Organization Global Health Observatory and the WHO Non-Communicable Disease Strategy. As part of this review, we identified the existence of national NCD strategies and whether CKD was included as part of an NCD strategic plan or addressed directly in a stand-alone policy. The existence and type of CKD-specific policies were also evaluated. We assessed specific kidney conditions (non-dialysis CKD, dialysis, and transplantation) covered by national policies, where available. Finally, government recognition of CKD as a priority in policy documents and the presence of national advocacy groups were also assessed. Data from desk research were supplemented by country-specific survey data.

### Country survey

We conducted a survey as an online questionnaire. The survey was administered electronically to representatives of all 182 countries with ISN affiliate societies between July and October 2018. We identified three key opinion leaders from each country: (1) a leader or president of a nephrology society, (2) a leader of a consumer representative organization, and (3) a policymaker, thus ensuring a diverse representation of perspectives from survey responders. Invitations were sent to key opinion leaders in each country to participate in the survey (available in English, French and Spanish), which included a link to the survey’s online portal (www.redcapcloud.com).

### Patient and public involvement

Patient care organisations (kidney foundations, patients’ associations) were involved in the development of the survey. The following organisations were involved in the survey, and their representatives were also selected to respond to the survey: European Kidney Patients’ Federation, International Federation of Kidney Foundations, Kidney Foundation of Canada, Kidney Health Australia and the US National Kidney Foundation.

### Statistical analysis

The data are presented as numbers (percentages) for categorical variables, means and standard deviations for normally distributed data, and medians with interquartile ranges, or medians with minimum and maximum values for non-normally distributed data. Survey data were analyzed and stratified based on the 4 World Bank income groups (low, low-middle, middle, and high-income) and the 10 ISN regions (Africa, Eastern and Central Europe, Latin America and the Carribbean, Middle East, NIS and Russia, North America, North and East Asia, Oceania and South East Asia, South Asia, and Western Europe). The results of the online survey were reported in accordance with the Checklist for Reporting Results of Internet E-Surveys (CHERRIES) guidelines.^28^ The data were analyzed using Stata 14 software (Stata Corporation).

## Results

Out of 182 invited countries, 160 (88%) participated in the GKHA survey, comprising 97.8% of the world’s population. One hundred and fifty-four countries responded to questions about national NCD and CKD strategies. Non-respondent countries were evenly distributed across regions and income groups and mostly represented smaller countries. Full details on response rates and population coverage of the survey have been previously published.

### National NCD and CKD-specific strategies

Seventy-four (47%) countries had an established national NCD strategy, with substantial variability across regions. Most countries in North America, North and East Asia, Oceania and South East Asia had a coordinated strategy for addressing NCDs (67, 71 and 80%, respectively), whilst only 27, 29 and 34% of countries in the Middle East, South Asia, and Africa, respectively, addressed NCDs through a national strategy (Table 1). 32% and 37% of low- and low-middle income countries, respectively, had a national NCD strategy (Table 1). The likelihood of having a national strategy increased across increasing World Bank income classification groups (S1 Fig). A national strategy was under development in 21 (14%) countries, whilst 42 (27%) had neither a strategy in place nor in development. Across all regions, only a minority of countries had a specific strategy for improving CKD care, mostly incorporated within existing NCD strategies (S2 Fig).

**Table 1:** Existence of a national strategy for NCDs. Numbers represent countries (%).

Location	National non-communicable chronic disease strategy				
	Yes	Under development	No	Unknown	n
**Overall**	73(47)	21(14)	42(27)	18(12)	154
**ISN regions:**					
• Africa	14(34)	5(12)	16(39)	6(15)	41
• Eastern & Central Europe	9(47)	4(21)	5(26)	1(5)	19
• Latin America & the Caribbean	10(56)	3(17)	4(22)	1(6)	18
• Middle East	3(27)	1(9)	6(55)	1(9)	11
• NIS & Russia	2(29)	2(29)	2(29)	1(14)	7
• North America	6(67)	1(11)	1(11)	1(11)	9
• North & East Asia	5(71)	0(0)	1(14)	1(14)	7
• Oceania & South East Asia	12(80)	1(7)	1(7)	1(7)	15
• South Asia	2(29)	3(43)	2(29)	0(0)	7
• Western Europe	10(50)	1(5)	4(20)	5(25)	20
**World Bank Groups:**					
• Low income	7(32)	3(14)	9(41)	3(14)	22
• Lower-middle income	13(37)	9(26)	9(26)	4(11)	35
• Upper-middle income	18(44)	6(15)	13(32)	4(10)	41
• High income	35(63)	3(5)	11(20)	7(13)	56

### Existence and type of CKD-specific policies

The existence of CKD-specific policies, both at national and regional levels, varied substantially according to country income (Table 2). Overall, 53 (34%) countries reported the existence of CKD-specific policies, with no low-income countries and 29% of low-middle and upper-middle income countries having CKD-specific policies (Table 2). More than half of countries in North and East Asia and Eastern and Central Europe had CKD-specific policies (57 and 58%, respectively) compared to Russia and NIS and Africa (0 and 15%, respectively). For those with CKD-specific policies, almost all were national policies with a small proportion of countries in upper-middle and high-income groups having both national and regional policies (S3 Fig).

**Table 2:** Existence of CKD-specific policies around the world. Numbers represent countries (%).

Location	CKD-specific policies			
	Yes	No	Unknown	n
**Overall**	53(34)	94(61)	7(5)	154
**ISN regions:**				
• Africa	6(15)	34(83)	1(2)	41
• Eastern & Central Europe	11(58)	8(42)	0(0)	19
• Latin America & the Caribbean	7(39)	10(56)	1(6)	18
• Middle East	4(36)	7(64)	0(0)	11
• NIS & Russia	0(0)	6(86)	1(14)	7
• North America	3(33)	4(44)	2(22)	9
• North & East Asia	4(57)	3(43)	0(0)	7
• Oceania & South East Asia	8(53)	7(47)	0(0)	15
• South Asia	1(14)	6(86)	0(0)	7
• Western Europe	9(45)	9(45)	2(10)	20
**World Bank Groups:**				
• Low income	0(0)	22(100)	0(0)	22
• Lower-middle income	10(29)	24(69)	1(3)	35
• Upper-middle income	12(29)	26(63)	3(7)	41
• High income	31(55)	22(39)	3(5)	56

### Specific kidney conditions covered by national policies

**Fig 1:**
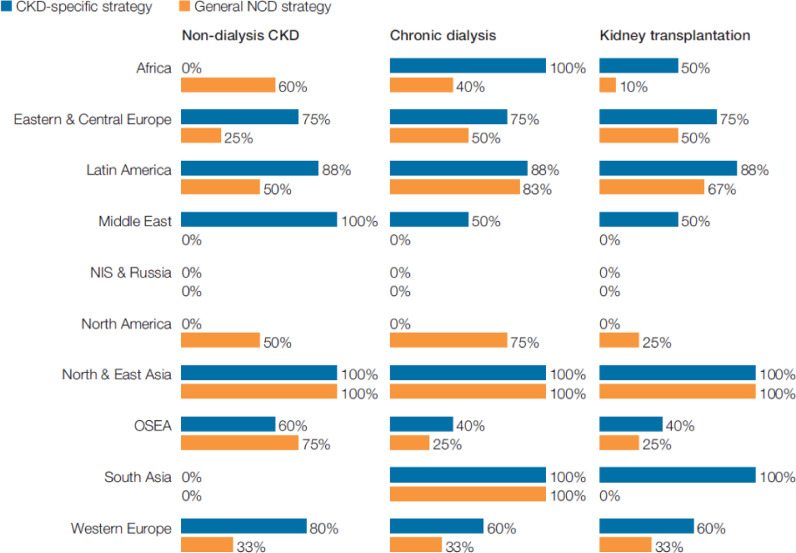
Kidney conditions covered by CKD-specific and general NCD strategies.

Dialysis and transplantation were more frequently covered in CKD-specific national policies compared to general NCD policies (Fig 1). However, dialysis and transplantation were not universally covered in CKD-specific national policies in many regions (Fig 1). Non-dialysis CKD care was not universally covered in CKD-specific or general NCD national policies outside of North and East Asia. No countries in South Asia addressed non-dialysis CKD care in CKD-specific or general NCD national policies, whilst no countries in Africa addressed non-dialysis CKD care through CKD-specific policies (Fig 1).

### Government support and advocacy group for AKI, CKD, and kidney failure treatment and prevention

**Fig 2:**
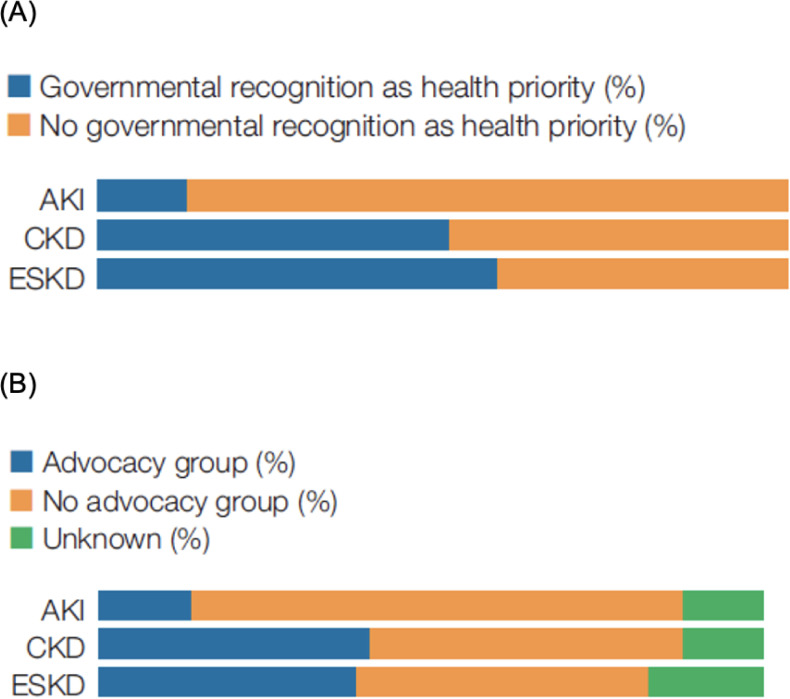
Government recognition as a priority (A) and availability of an advocacy group (B) for AKI, CKD, and kidney failure treatment and prevention.

Governments more frequently recognized CKD and kidney failure as health priorities compared to acute kidney injury (Fig 2). Overall, 79 (51%) countries reported that governments recognized CKD as a health priority. This varied substantially across regions with 27% of low-income and 43% of low-middle income countries recognizing CKD as a health priority, compared to 63 and 57% of upper middle- and high-income countries, respectively (Table 3). Like governmental priorities for specific kidney diseases, advocacy groups for acute kidney injury were less common than for CKD and kidney failure (Fig 2). However, the proportions of countries in which advocacy organizations existed for CKD and/or kidney failure were generally low, particularly in low- and low-middle income countries (Table 3).

**Table 3:** Government prioritization of, and presence of advocacy groups for AKI, CKD, and kidney failure treatment and prevention around the world. Numbers represent countries (%).

Location	Governmental recognition of CKD as a health priority		Presence of advocacy group for CKD			Governmental recognition of AKI as a health priority		Presence of advocacy group for AKI			Governmental recognition of kidney failure/KRT as a health priority		Presence of advocacy group for kidney failure/KRT		
	Yes	No	Yes	No	Unknown	Yes	No	Yes	No	Unknown	Yes	No	Yes	No	Unknown
Overall	79(51)	75(49)	63(41)	72(47)	19(12)	89(58)	65(42)	60(39)	68(44)	26(17)	89(58)	65(42)	60(39)	68(44)	26(17)
ISN regions:															
• Africa	20(49)	21(51)	12(29)	23(56)	6(15)	15(37)	26(63)	12(29)	22(54)	7(17)	15(37)	26(63)	12(29)	22(54)	7(17)
• Eastern & Central Europe	10(53)	9(47)	9(47)	9(47)	1(5)	14(74)	5(26)	8(42)	9(47)	2(11)	14(74)	5(26)	8(42)	9(47)	2(11)
• Latin America & the Caribbean	12(67)	6(33)	10(56)	7(39)	1(6)	8(44)	10(56)	9(50)	7(39)	2(11)	8(44)	10(56)	9(50)	7(39)	2(11)
• Middle East	4(36)	7(64)	3(27)	7(64)	1(9)	8(73)	3(27)	1(9)	9(82)	1(9)	8(73)	3(27)	1(9)	9(82)	1(9)
• NIS & Russia	2(29)	5(71)	1(14)	3(43)	1(14)	6(86)	1(14)	2(29)	4(57)	1(14)	6(86)	1(14)	2(29)	4(57)	1(14)
• North America	7(78)	2(22)	6(67)	3(33)	1(11)	6(67)	3(33)	5(56)	4(44)	0(0)	6(67)	3(33)	5(56)	4(44)	0(0)
• North & East Asia	5(71)	2(29)	5(71)	2(29)	3(43)	7(100)	0(0)	6(86)	1(14)	0(0)	7(100)	0(0)	6(86)	1(14)	0(0)
• Oceania & South East Asia	7(47)	8(53)	9(60)	5(33)	1(7)	8(53)	7(47)	6(40)	4(27)	5(33)	8(53)	7(47)	6(40)	4(27)	5(33)
• South Asia	2(29)	5(71)	4(57)	3(43)	1(14)	4(57)	3(43)	4(57)	2(29)	1(14)	4(57)	3(43)	4(57)	2(29)	1(14)
• Western Europe	10(50)	10(50)	4(20)	10(50)	3(15)	13(65)	7(35)	7(35)	6(30)	7(35)	13(65)	7(35)	7(35)	6(30)	7(35)
World Bank Groups:															
• Low income	6(27)	16(73)	5(23)	16(73)	2(9)	5(23)	17(77)	4(18)	14(64)	4(18)	5(23)	17(77)	4(18)	14(64)	4(18)
• Lower-middle income	15(43)	20(57)	14(40)	16(46)	5(14)	20(57)	15(43)	12(34)	14(40)	9(26)	20(57)	15(43)	12(34)	14(40)	9(26)
• Upper-middle income	26(63)	15(37)	20(49)	15(37)	5(12)	25(61)	16(39)	20(49)	16(39)	5(12)	25(61)	16(39)	20(49)	16(39)	5(12)
• High income	32(57)	24(43)	24(43)	25(45)	7(13)	39(70)	17(30)	24(43)	24(43)	8(14)	39(70)	17(30)	24(43)	24(43)	8(14)

## Discussion

In this comprehensive multinational survey of kidney care in 160 countries, we made three main observations with regards to national health policies and strategies for addressing CKD. Firstly, less than half of countries had an established NCD strategy, and overall, only a third of countries had a CKD specific policy, with substantial variation across income levels and regions on both accounts. Secondly, where CKD-specific policies existed, non-dialysis CKD care was variably addressed despite this population encompassing the vast majority of people living with CKD. Thirdly, CKD was identified as a health priority by governments in only half of countries overall, and even less frequently in low and low-middle income countries, where the largest increases in disease burden are occurring. Taken together, these findings indicate that CKD remains substantially underprioritized globally relative to its current and projected burden on individuals and health systems.

Best available data indicate that CKD has a major effect on global health, both as a direct cause of morbidity and mortality and as a risk multiplier for cardiovascular disease [12]. A large proportion of the burden of CKD is concentrated in low- and middle-income countries, particularly Oceania, sub-Saharan Africa and Latin America [3]. It is these same countries that are least equipped to provide equitable access to treatment for kidney failure due to the enormous cost of dialysis and kidney transplantation [6, 13]. In 2010, only half of people worldwide who required dialysis were able to access this life-sustaining treatment, predominantly due to cost [5]. COVID-19 has further exposed and amplified healthcare inequalities, [14] with people living with CKD, especially those with kidney failure, at much greater risk of serious morbidity and mortality [15]. Our findings indicate a major discrepancy between the burden of CKD in many countries and national health policy settings as evidenced by the absence of CKD specific policies and government recognition of CKD as a national priority.

Given the potential for national policies and statements to influence healthcare priorities, our findings argue for urgent attention from governments and policy makers. Indeed, developing and implementing national CKD-specific policies, or integrating CKD into existing or planned NCD strategies, may assist governments and key stakeholders to evaluate and identify gaps in care and advocate for health system improvement. Where they exist, CKD registries provide critical insights, for example temporal trends in quality of care, changes in epidemiology over time and health service utilisation. This information can serve several important purposes including informing the development of CKD policies and guidelines, benchmarking implementation strategies, and serving as a tool for advocacy to urge policy makers to tackle primary prevention of NCDs and reform towards Universal Health Coverage. Multiple registries across different health systems indicate that the use of therapies with proven benefits in CKD remains unacceptably low; in India; less than half of patients with mild-moderate CKD received renin-angiotensin system blockade and statins, with similar findings observed in high-income countries [16–18]. Therefore, when coupled with robust health information systems, CKD-specific policies may help to accelerate implementation science in CKD by providing a local standard by which to evaluate the quality of kidney care–a key priority identified by the ISN to improve global kidney health [19]. Additionally, revaluating the role of screening and early detection of CKD within national NCD and CKD policies will be important as new therapies become increasingly available [20]. Of note, sodium glucose cotransporter 2 inhibitors, are now included in the World Health Organization Essential Medicines List. If access to these agents is expanded in an affordable manner, there is the potential to substantially improve outcomes and influence the epidemiology of CKD (and cardiovascular disease) in many in low- and middle-income countries [21–25]. In this respect, integration of CKD into existing or planned national NCD policies and strategies may be less daunting for policy makers and strengthen commitment towards addressing common risk factors such as diabetes and hypertension, and coexisting conditions such as cardiovascular disease and heart failure.

Whilst international clinical practice guidelines, position papers and scientific statements provide overarching recommendations about best practice, country-specific policies and strategies play an important role in interpreting these recommendations in the context of local disease prevalence patterns and resource availability. For example, strategies and polices for addressing CKD of unknown aetiology are most relevant to Latin America, whereas specific strategies for diabetic kidney disease may be more relevant in parts of Oceania and South East Asia [26, 27]. The availability of kidney replacement therapy also varies widely across regions, and clearly has important implications for CKD-specific national policies [28, 29].

However, the development and implementation of national NCD and CKD-specific strategies and policies in isolation is unlikely to substantially improve kidney health. Supporting countries to develop CKD-specific policies must be coupled with much more widespread testing for albuminuria in high-risk individuals, such as those with type 2 diabetes (which remains poor even in high income countries), [30] greater access to proven therapies, [31] and the establishment and refinement of health information systems to evaluate quality of care [32]. Efforts to address shared risk factors for other NCDs, such as hypertension and diabetes, are likely to result in the greatest gains, and should be a major focus in all national NCDs policies [6].

This study has several important strengths. The ISN GKHA is one of the largest ever health-related country capacity reviews undertaken and all regions and World Bank income levels were well represented in the study. The combined use of desk research and survey responses from key on-the-ground stakeholders and national and regional leaders ensured the data were accurate. The survey was based on a well validated framework for assessing healthcare capacity based on the World Health Organisation health system building blocks. However, our findings should be interpreted in light of some limitations. While the survey was completed by national leaders, key consumer representative organizations and policymakers, responses may not have captured variations within large countries where policies may have varied across jurisdictions. Responses may also have been affected by respondents’ knowledge and experience and been in part subjective. More granular details on the nature of CKD-specific policies, including variations in local NCD or CKD-specific policies (e.g. local pathways of care) within countries were not collected given the size and scope of the GKHA. The GKHA also did not collect information on the implementation of NCD or CKD specific policies, both at a national or local level, which highlights the value of studies that may be able to summarize variations in adherence to guidelines and outcomes. Such implementation studies will be critical to ensure the translation of evidence and guidelines to improve population health for CKD and related NCDs. Nevertheless, this study provides the most comprehensive global overview to date of national health policies and strategies for addressing CKD, which may serve as an advocacy tool to promote the development of national strategies and policies. Future iterations of the GKHA can also be used to gauge progress over time.

In summary, the existence of CKD-specific policies, and a national NCD strategy more broadly, varied substantially across different regions around the world but was suboptimal overall. Greater recognition of CKD within national health policies and NCD strategies is critical to improving kidney healthcare globally.

## Supporting information

S1 Fig Existence of a national strategy for NCDs. (EPS)

S2 Fig Existence of a national strategy for improving CKD care. (EPS)

S3 Fig (A) Existence and (B) type of CKD-specific policies. (EPS)
